# Bronchial Ligation as a Temporizing Procedure for Hemoptysis Caused by Acute Type B Aortic Dissection Rupturing into the Lung with Subsequent TEVAR: A Case Report

**DOI:** 10.3390/reports9030306

**Published:** 2026-09-11

**Authors:** Ryo Maekawa, Shun-Ichiro Sakamoto, Atsushi Hiromoto, Kenji Suzuki, Yosuke Ishii

**Affiliations:** 1 Department of Cardiovascular Surgery, Nippon Medical School Musashikosugi Hospital, Kawasaki 211-8533, Japan; 2 Department of Cardiovascular Surgery, Nippon Medical School, Tokyo 113-8603, Japan

**Keywords:** acute type B aortic dissection, aortobronchial fistula, bronchial ligation, case report

## Abstract

**Background and Clinical Significance:** Hemoptysis caused by acute type B aortic dissection rupturing into the lung is rare but life-threatening; **Case Presentation:** An 81-year-old man receiving maintenance hemodialysis developed hemoptysis due to an aortobronchial fistula involving the left upper lobe. The ulcer-like projection was located on the greater curvature of the distal aortic arch and enlarged during the clinical course. Immediate thoracic endovascular aortic repair (TEVAR) and open repair were initially considered unsuitable because of hostile access through a previous abdominal aortic graft, a shaggy aorta, and severe cardiac comorbidities. Left upper lobe bronchial ligation was performed as a temporizing airway-isolation procedure and initially controlled airway bleeding. However, recurrent hemoptysis occurred after further enlargement of the aortic lesion and development of a new fistula involving the left lower lobe. Emergency TEVAR was then performed through the previous abdominal aortic prosthetic graft, and no further hemoptysis occurred; **Conclusions:** Bronchial ligation may temporarily control life-threatening airway bleeding when immediate aortic repair is not feasible, but strict surveillance and readiness for subsequent aortic intervention remain essential.

## 1. Introduction and Clinical Significance

Hemoptysis caused by thoracic aortic disease is rare but potentially fatal [1]. Rupture of a thoracic aortic aneurysm, pseudoaneurysm, or acute aortic dissection into the lung or bronchial tree may present as intermittent or massive hemoptysis and can rapidly lead to airway obstruction or exsanguination [2,3]. Aortobronchial fistula and aortic rupture into the lung have been treated by open aortic repair, thoracic endovascular aortic repair, lung resection, or staged and hybrid procedures [4]. The fundamental principle of treatment is control of the aortic lesion [1,4].

However, immediate aortic repair may be difficult in patients with hostile access routes, previous aortic reconstruction, shaggy aorta, severe cardiac dysfunction, or other high-risk conditions. This problem is particularly important in acute aortic dissection, in which false lumen pressurization, ulcer-like projection (ULP) enlargement, and local rupture may progress over a short period. In such patients, temporary control of airway bleeding may be required to prevent fatal hemoptysis and aspiration while maintaining the possibility of subsequent definitive aortic treatment. The role of nondefinitive airway-directed surgical procedures in this setting remains poorly defined.

We report a case of acute type B aortic dissection rupturing into the lung that caused hemoptysis in a high-risk patient. Because immediate thoracic endovascular aortic repair and open aortic repair were considered unsuitable, bronchial ligation was performed as a temporizing procedure, followed by subsequent thoracic endovascular aortic repair. This case suggests that bronchial ligation may be an option for temporary control of life-threatening hemoptysis caused by aortic rupture into the lung when immediate aortic repair is not feasible.

## 2. Case Presentation

**Figure 1:**
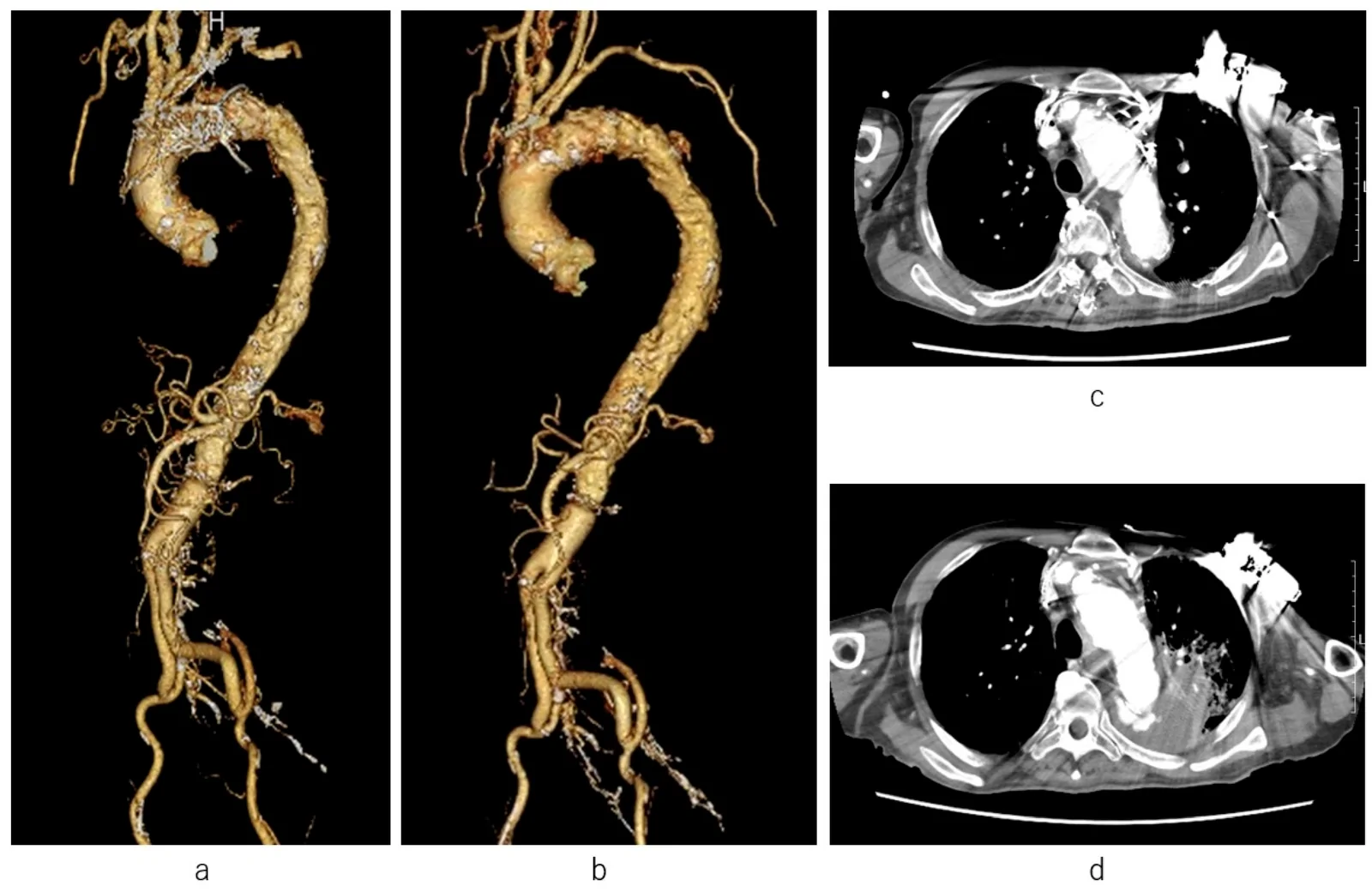
Contrast-enhanced computed tomography findings before and after the onset of hemoptysis. (**a**,**c**) Initial computed tomography showed acute type B aortic dissection with a small ulcer-like projection and no pulmonary infiltrative shadow in the left upper lobe. (**b**,**d**) After the onset of hemoptysis, the ulcer-like projection enlarged, with contrast inflow into the thrombosed false lumen. An infiltrative shadow appeared in the left upper lobe, suggesting pulmonary hemorrhage due to an aortobronchial fistula involving the peripheral bronchus of the left upper lobe.

An 81-year-old man was transported to our hospital with a chief complaint of lower back pain. Contrast-enhanced computed tomography (CT) revealed acute type B aortic dissection. The dissection was classified as DeBakey type IIIa, and no evidence of malperfusion was observed. Although a shaggy aorta was present, the ulcer-like projection (ULP) was initially small; therefore, conservative management was selected (Figure 1a,c).

After transfer to the general ward, the patient developed hemoptysis on day 18 after the onset of aortic dissection. Contrast-enhanced CT showed enlargement of the known ULP on the greater curvature of the distal aortic arch, which appeared to act as an entry tear. The maximum external diameter of the ULP at this time was approximately 20 mm. Contrast inflow into the thrombosed false lumen was observed, and recanalization of the false lumen was accompanied by enlargement of the false lumen diameter. In addition, an infiltrative shadow was observed in the left upper lobe. No active airway bleeding was observed immediately after tracheal intubation, and upper gastrointestinal endoscopy confirmed the absence of an aortoesophageal fistula. Based on the CT findings and clinical presentation, hemoptysis was diagnosed as resulting from an aortobronchial fistula involving the peripheral bronchus of the left upper lobe (Figure 1b,d).

The patient was receiving maintenance hemodialysis and had a cardiac resynchronization therapy defibrillator (CRT-D) implanted for chronic heart failure, complete left bundle branch block, and sustained ventricular tachycardia. He had also previously undergone prosthetic graft replacement for an abdominal aortic aneurysm, and both graft limbs had been anastomosed end-to-side to the external iliac arteries. Therefore, open surgical repair was considered to be associated with prohibitive risk. Although a formal surgical risk score was not calculated, the risk was judged to be prohibitive in a multidisciplinary discussion because of maintenance hemodialysis, chronic heart failure requiring CRT-D implantation, a history of sustained ventricular tachycardia, acute aortic dissection, shaggy aorta, and previous abdominal aortic graft reconstruction. TEVAR was also considered technically difficult because of the previous abdominal aortic graft reconstruction, anticipated resistance at the graft limb and external iliac artery anastomosis, and the risk of embolic complications and retrograde type A aortic dissection associated with manipulation of a shaggy arch. Because no persistent bleeding was observed after tracheal intubation, initial management under mechanical ventilation was selected. Subsequently, to prevent recurrent hemoptysis, bronchial ligation was planned and performed 8 days after the onset of hemoptysis.

**Figure 2:**
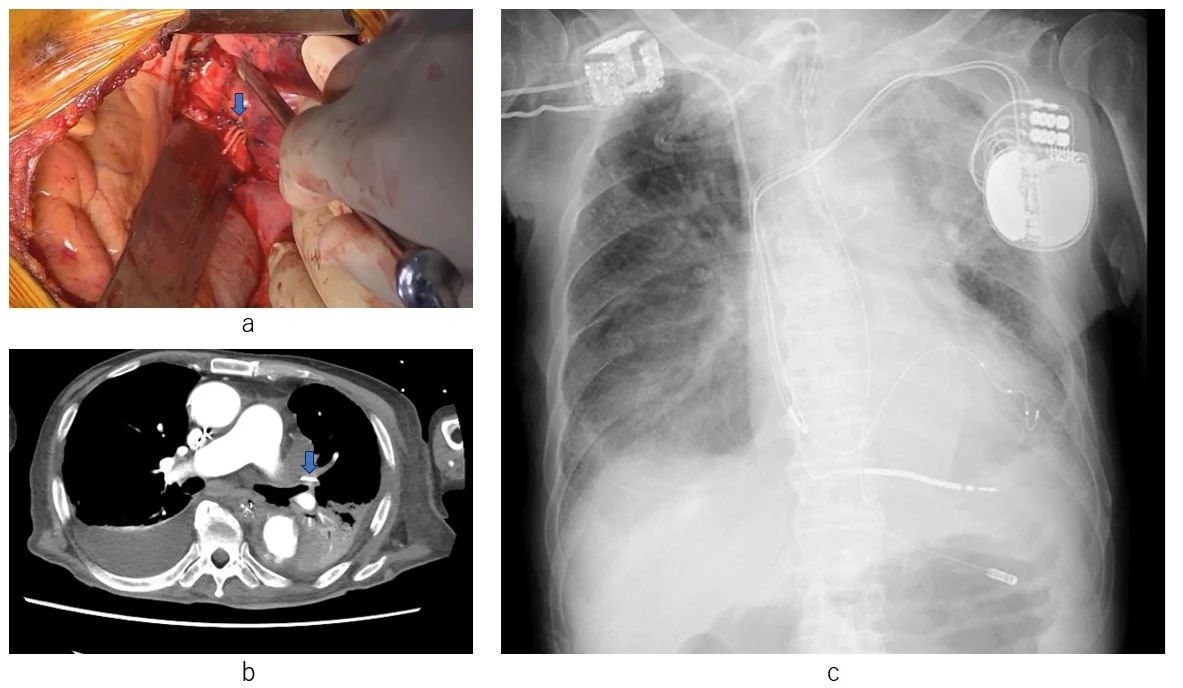
Bronchial ligation and postoperative findings. (**a**) Intraoperative photograph showing ligation of the left upper lobe bronchus under thoracoscopic guidance. The blue arrow indicates a hemoclip placed to reinforce the distal knot of the ligature. (**b**) Postoperative contrast-enhanced computed tomography showing a hemoclip marking the site of the ligated left upper lobe bronchus. The arrow indicates the hemoclip. (**c**) Chest radiograph after bronchial ligation showing good expansion of the left lung, except for residual atelectasis of the left upper lobe.

The operation was performed with the patient in the right lateral decubitus position under one-lung ventilation. A left thoracotomy was performed through the fourth intercostal space. After the pulmonary vein was divided at the hilum to obtain an adequate operative field, the left upper lobe bronchus was ligated under thoracoscopic guidance, and the distal knot of the ligature was reinforced with a hemoclip (Figure 2a,b). This maneuver was not intended as part of lobectomy; rather, it was required to secure a sufficient operative field around the hilar structures and to expose the left upper lobe bronchus safely for reliable ligation. Postoperatively, airway bleeding ceased, and the left lung remained well expanded, except for residual atelectasis of the left upper lobe (Figure 2c). The nonventilated left upper lobe distal to the bronchial ligation was managed conservatively under perioperative antibiotic therapy. No fever, lung abscess, or uncontrolled inflammatory response attributable to the nonventilated left upper lobe occurred, and the residual atelectatic lobe was considered to have followed an organizing course. Tracheostomy was performed 16 days after the onset of hemoptysis. Weaning from mechanical ventilation was then attempted, and the patient was successfully liberated from the ventilator 34 days after the onset of hemoptysis.

**Figure 3:**
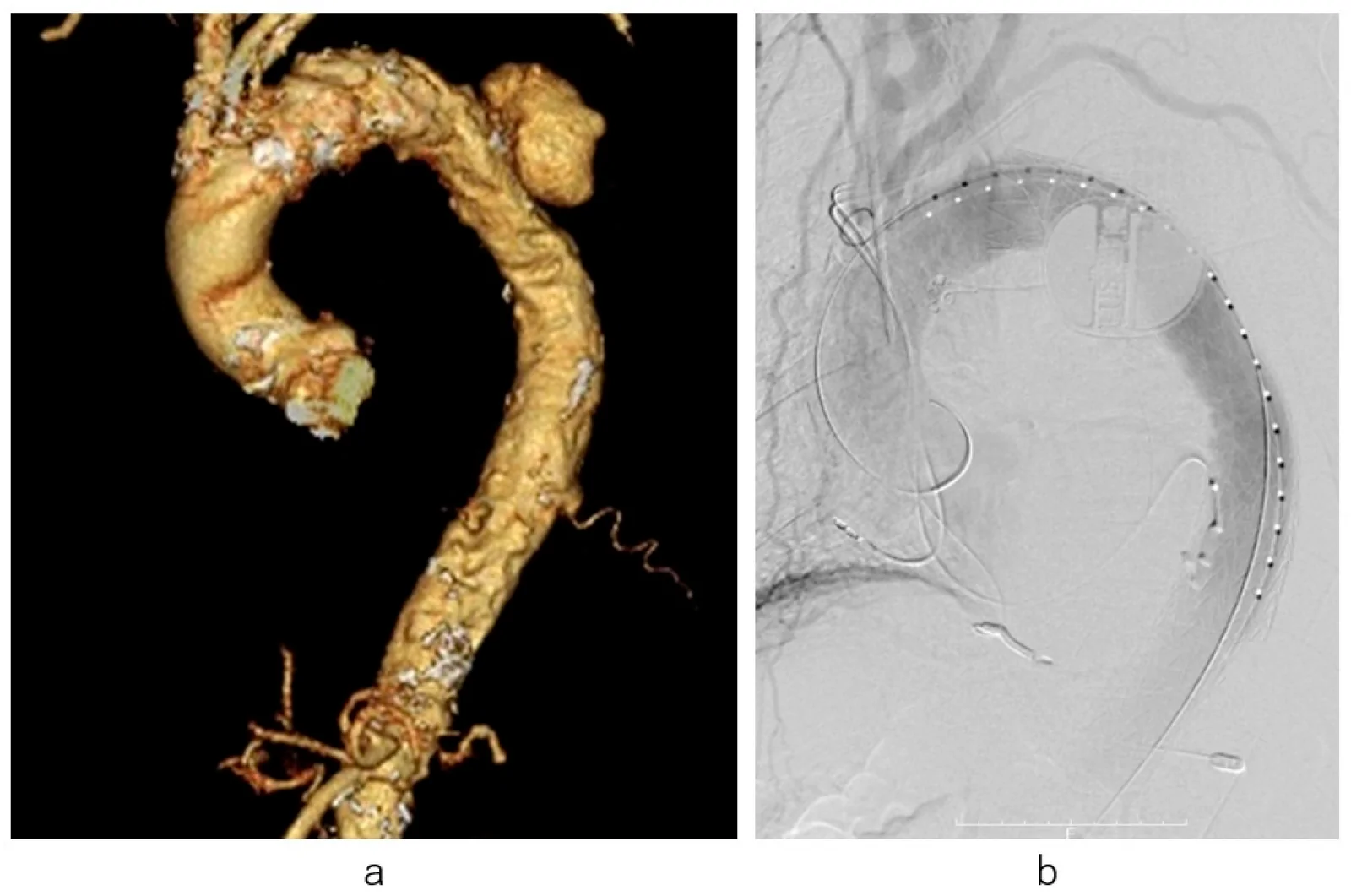
Recurrent rupture and subsequent thoracic endovascular aortic repair. (**a**) Three-dimensional computed tomography after recurrent hemoptysis showed further enlargement of the ulcer-like projection and descending thoracic aorta. A new aortobronchial fistula involving the peripheral bronchus of the left lower lobe was suspected. (**b**) Completion angiography after thoracic endovascular aortic repair showed exclusion of the aortic lesion without endoleak.

However, recurrent hemoptysis occurred the following day. Bronchoscopy demonstrated bleeding from a peripheral bronchus of the left lower lobe. Contrast-enhanced CT showed further enlargement of the ULP at the same site on the greater curvature of the distal aortic arch, with a maximum external diameter of approximately 50 mm, as well as enlargement of the descending thoracic aorta. A new aortobronchial fistula involving the peripheral bronchus of the left lower lobe was diagnosed (Figure 3a). Emergency TEVAR was performed. The left common femoral artery was exposed through an oblique groin incision, and a purse-string suture was placed. A 4-Fr sheath was inserted into the right common femoral artery for angiography, and a 5-Fr sheath was inserted into the left common femoral artery. A guidewire was advanced to the ascending aorta and exchanged for a Lunderquist wire. A 22-Fr DrySeal sheath (W. L. Gore & Associates, Flagstaff, AZ, USA) was advanced through the left common femoral artery. Although resistance was encountered around the anastomotic region between the previous prosthetic graft limb and the external iliac artery, passage of the sheath was possible. Systemic heparinization was performed to maintain an activated clotting time of approximately 200 s.

Two GORE TAG Conformable Thoracic Stent Grafts Active Control System (cTAG AC; W. L. Gore & Associates) were deployed. A 34 mm × 34 mm × 20 cm cTAG AC was first placed proximally with the proximal landing zone in zone 3 while avoiding excessive manipulation of the shaggy arch. Because the distal edge of the first stent graft was located just proximal to the aortic lesion, an additional 34 mm × 34 mm × 10 cm cTAG AC was deployed distally with approximately 5 cm of overlap. Balloon molding was not performed because of concern for retrograde type A aortic dissection. Completion angiography confirmed exclusion of the aortic lesion without major endoleak (Figure 3b). Before removing the sheath, angiography confirmed no injury at the prosthetic graft anastomosis or access route. The left common femoral artery was closed with the purse-string suture and additional sutures.

After TEVAR, the patient had no further bleeding episodes, including hemoptysis, and his vital signs remained stable. He received antibiotic therapy for postoperative pneumonia. Considering the risk of recurrent airway bleeding, routine systemic anticoagulation or antiplatelet therapy was not initiated after TEVAR. Maintenance hemodialysis was continued three times weekly through the superficialized artery, with nafamostat mesilate used only during dialysis as the anticoagulant. Continuous hemodiafiltration was not required. The patient was successfully liberated from mechanical ventilation 7 days after TEVAR. All diagnostic evaluations and procedures described in this report, including bronchial ligation and TEVAR, were performed at our institution. He remained afebrile with stable vital signs and was transferred to another hospital for continued rehabilitation 36 days after TEVAR. Active treatment was initially pursued according to the wishes of the patient and his family; however, after transfer for rehabilitation, goals of care were re-discussed because of progressive disuse syndrome. Four months after transfer to the rehabilitation hospital, the patient died of heart failure. No recurrent hemoptysis or aortic rupture was reported after TEVAR.

## 3. Discussion

Open aortic repair with graft replacement, with or without lung resection, is considered the most definitive treatment for aortobronchial fistula or aortic rupture into the lung because it allows direct repair of the aortic lesion and management of the bronchopulmonary end. However, open repair is highly invasive, and conventional open repair for aortobronchial fistula has been associated with reported surgical mortality rates of 15% to 41% [5]. TEVAR has emerged as a less invasive alternative and provides favorable early outcomes. In a systematic review, Canaud et al. reported a technical success rate of 93.2% and a 30-day mortality rate of 5.9% after TEVAR for aortobronchial fistula [4]. However, aortic-related mortality and recurrence of aortobronchial fistula during follow-up were reported in 14.3% and 11.1% of patients, respectively [4]. Furthermore, the long-term durability of TEVAR alone remains uncertain, because the fistulous tract, pulmonary hematoma, infected space, or contaminated tissue is not directly removed [5]. Therefore, TEVAR may be effective for early bleeding control and stabilization, but careful long-term follow-up is mandatory.

Several case reports have described hemoptysis associated with acute aortic dissection or aortobronchial fistula; however, the therapeutic strategies differed from that used in the present case. Minegishi et al. reported hemoptysis caused by Stanford type A acute aortic dissection with posterior mediastinal hematoma extending along the right pulmonary artery, which was successfully treated by emergency ascending aortic replacement [2]. Sakai et al. reported acute life-threatening massive hemoptysis due to an aortobronchial fistula caused by thoracic aortic dissection, in which thick wedge resection of the lung with hematoma using a stapler was performed to control hemoptysis, followed by graft replacement of the distal arch and descending thoracic aorta [6]. In contrast to these reports, immediate open aortic repair or TEVAR was initially considered unsuitable in our patient because of hostile access, shaggy aorta, and severe comorbidities. Therefore, bronchial ligation was selected as a temporizing airway-isolation procedure rather than as part of definitive aortic repair.

In the present case, TEVAR was initially considered technically challenging because the patient had previously undergone abdominal aortic replacement with a Y-shaped prosthesis, with both graft limbs anastomosed end-to-side to the external iliac arteries. Shimizu et al. reported that standard femoral access for TEVAR was used significantly less often in patients with prior abdominal or iliac prosthetic reconstruction than in those without such history [7]. In our case, this concern was confirmed intraoperatively during emergency TEVAR, because resistance was encountered around the previous graft limb and external iliac artery anastomosis during advancement of the 22-Fr DrySeal sheath. In addition, the proximal landing zone showed severe atherosclerotic change, raising concern about embolic complications during device manipulation and stent-graft-induced aortic injury. Retrograde type A aortic dissection is a rare but catastrophic complication after TEVAR, and Chen et al. reported a pooled incidence of 2.5% and mortality of 37.1% in a systematic review [8]. The risk was higher in patients treated for dissection, especially acute dissection, than in those treated for aneurysm [8]. These anatomical and procedural concerns contributed to the initial decision to avoid TEVAR and later influenced the decision to avoid balloon molding during emergency TEVAR. Thus, the initial decision was not based on a preference for bronchial ligation over TEVAR, but on the judgment that immediate TEVAR itself carried an unacceptably high procedural risk at that time. After recurrent hemoptysis and further enlargement of the ulcer-like projection, the risk-benefit balance changed, and TEVAR was performed as a salvage procedure despite the anticipated access difficulty and aortic risks.

Under these circumstances, bronchial ligation was performed to prevent recurrent hemoptysis and protect the remaining airway from blood aspiration. Isolation of the bleeding airway is a key principle in the management of massive hemoptysis. Selective intubation, bronchial blockers, balloon tamponade, and endobronchial occlusion have been used to prevent blood from entering the contralateral lung and central airway [9]. Endobronchial occlusion using a bronchial blocker or plug may represent another less invasive option for airway isolation in selected patients with massive hemoptysis. In the present case, bronchoscopic plug occlusion was not attempted. However, the bleeding source was presumed to involve a peripheral bronchus of the left upper lobe associated with aortic rupture into the lung, and prolonged airway isolation was expected to be necessary because immediate aortic repair was considered unsafe. Therefore, surgical ligation of the left upper lobe bronchus was selected to provide more reliable and durable isolation of the responsible lobe. In contrast, surgical bronchial ligation has rarely been described for hemoptysis caused by aortic rupture into the lung. In this case, ligation of the responsible bronchus was intended to provide durable isolation of the bleeding lobe and prevent airway obstruction due to recurrent bleeding. To the best of our knowledge, bronchial ligation as a temporizing procedure for hemoptysis caused by acute type B aortic dissection rupturing into the lung has not been previously reported.

The subsequent clinical course illustrates the limitation of bronchial ligation. This procedure does not treat the underlying aortic pathology and should be regarded as a temporizing procedure rather than definitive therapy. In the present case, although airway bleeding was initially controlled after left upper lobe bronchial ligation, subsequent recanalization and enlargement of the false lumen resulted in a new fistula involving the peripheral bronchus of the left lower lobe, necessitating emergency TEVAR. Bronchial ligation can temporarily isolate the bleeding airway and prevent fatal airway obstruction, but it cannot prevent progression of the dissected aorta or rupture into another pulmonary site. Therefore, even after successful control of hemoptysis, strict imaging follow-up, reassessment of vascular access, optimization of the patient’s general condition, and readiness for prompt TEVAR are essential.

## 4. Conclusions

Bronchial ligation may be considered as a temporizing option for life-threatening hemoptysis caused by acute type B aortic dissection rupturing into the lung when immediate TEVAR or open repair is not feasible. However, because this procedure does not address the underlying aortic pathology, strict imaging surveillance, reassessment of vascular access, and readiness for subsequent aortic intervention are essential.

## Data Availability

The data presented in this study are available on request from the corresponding author due to privacy and ethical restrictions.
